# Variable NF-κB pathway responses in colon cancer cells treated with chemotherapeutic drugs

**DOI:** 10.1186/1471-2407-14-599

**Published:** 2014-08-18

**Authors:** Temesgen Samuel, Khalda Fadlalla, Dominique N Gales, Balananda DK Putcha, Upender Manne

**Affiliations:** School of Veterinary Medicine, Pathobiology Department and TU Center for Cancer Research, Tuskegee University, Tuskegee, AL 36088 USA; Department of Pathology and Comprehensive Cancer Center, University of Alabama at Birmingham, Birmingham, AL 35294 USA

**Keywords:** Colon cancer, NF-κB, Camptothecin, Drug response, Cytokine, Chemokine

## Abstract

**Background:**

The nuclear factor kappa-light-chain-enhancer of activated B cells (NF-κB) signaling pathway is activated in cells exposed to various stimuli, including those originating on the cell surface or in the nucleus. Activated NF-κB signaling is thought to enhance cell survival in response to these stimuli, which include chemotherapy and radiation. In the present effort, we determined which anticancer drugs preferentially activate NF-κB in colon cancer cells.

**Methods:**

NF-κB reporter cells were established and treated with 5-fluorouracil (5-FU, DNA/RNA damaging), oxaliplatin (DNA damaging), camptothecin (CTP, topoisomerase inhibitor), phleomycin (radiomimetic), or erlotinib (EGFR inhibitor). The activation of NF-κB was assessed by immunofluorescence for p65 translocation, luciferase assays, and downstream targets of NF-κB activation (cIAP2, and Bcl-X_L_) were evaluated by immunoblotting, by ELISA (CXCL8 and IL-6 in culture supernatants), or by gene expression analysis.

**Results:**

Colon cancer cells responded variably to different classes of therapeutic agents, and these agents initiated variable responses among different cell types. CPT activated NF-κB in SW480 colon cancer cells in a dose-dependent manner, but not in HCT116 cells that were either wild-type or deficient for p53. In SW480 colon cancer cells, NF-κB activation by CPT was accompanied by secretion of the cytokine CXCL8, but not by up-regulation of the anti-apoptotic genes, cIAP2 or Bcl-X_L_. On the contrary, treatment of HCT116 cells with CPT resulted in up-regulation of CXCR2, a receptor for CXCL8, without an increase in cytokine levels. In SW480 cells, NF-κB reporter activity, but not cytokine secretion, was inhibited by SM-7368, an NF-κB inhibitor.

**Conclusion:**

The results show that, in response to cancer therapeutic agents, NF-κB activation varies with the cellular make up and that drug-induced NF-κB activation may be functionally uncoupled from anti-apoptotic outcomes found for other stimuli. Some cancer cells in a heterogeneous tumor tissue may, under therapeutic pressure, release soluble factors that have paracrine activity on neighboring cells that express the cognate receptors.

**Electronic supplementary material:**

The online version of this article (doi:10.1186/1471-2407-14-599) contains supplementary material, which is available to authorized users.

## Background

The outcomes of cancer therapy depend on various determinants that include tumor-intrinsic factors and inter-individual variation in drug response and metabolism. It is still not possible to predict with certainty the response of a given tumor to a particular chemotherapeutic agent. The core tenet of personalized cancer medicine is to identify subsets of patients who will favorably respond to a given therapy and to avoid non-beneficial drug exposure for those who may not respond [1–4]. The efficacy of chemotherapy, especially that of non-targeted agents, is hindered by dose-limiting toxicity and by the development of non-responsiveness. Although targeted agents are designed to reduce the off-target effects of chemotherapy, the development of resistance has hindered progress in cancer therapy and management [2, 5–7]. In line with the potential of personalized medicine, it is essential to identify the genetic, epigenetic, and adaptive characteristics of cancer cells and other cells in the microenvironment that contribute to response to both targeted and broad-acting drugs.

The NF-κB pathway is now a target for therapeutic development, primarily because of its role in chronic inflammatory states, which promote oncogenesis [8–11]. Moreover, experimental and association studies indicate the benefits of suppressing chronic inflammation in reducing the incidence of various types of cancers [12–17]. Moreover, the risk of cancer is higher among colitis patients, and chronic bacterial infection by *H. pylori* is linked to gastric cancer [18–23].

Nevertheless, the NF-κB mechanism, which contributes to the initiation and progression of cancer, is activated by anticancer drugs and radiation [24–27]. Such activation is clinically undesirable because cells may emerge as resistant, once they are relieved of the drug pressure, or may carry mutations that drive their aggressiveness. Cancer stem-like cells, which utilize the NF-κB pathway, may be responsible for resistance and for re-seeding of the tumor mass after initially effective chemotherapy or radiation [28–31].

The mechanisms through which drugs induce NF-κB activation, and how NF-κB-driven gene expression contributes to drug resistance or other functions, are not fully understood. Drug-induced damage to cancer cell DNA is thought to activate NF-κB through the protein IKK-gamma. DNA-damage activates ATM kinase, which in turn activates NF-κB essential modifier (NEMO), a component of the IKK complex that induces nuclear translocation of the p65/p50 transcription factor complex [24, 32, 33]. The determinants for drug-induced NF-κB activation and the function of activated NF-κB in this context remain to be elucidated.

In the present investigation, reporter cells that carry NF-κB response elements linked to the luciferase gene were used to examine the response of colon cancer cells to drugs. Activation of NF-κB by chemotherapeutic drugs and the downstream effects of the activation varied among cell lines and drug types. Moreover, in the colon cancer cells, the cytokine response was apparently uncoupled from expression of anti-apoptotic genes.

## Methods

### Cell lines and culture

SW480 human colon cancer cells were from American Type Cell Culture (ATCC, Manassas, VA; CCL-228, and CRL-2577). Wild-type and p53-null (p53-/-) HCT116 colon cancer cells were generous gifts from Dr. Bert Vogelstein (Johns Hopkins, Baltimore, MD). Both cell lines were grown in McCoy’s 5A culture medium (ATCC® 30-2007) containing 10% fetal bovine serum, penicillin (10,000 U/ml) and streptomycin (10 mg/ml).

### Drugs and reagents

TNFα, 5-FU, CPT, and phleomycin were purchased from Sigma Aldrich (St. Louis, MO); oxaliplatin and erlotinib were purchased from LC laboratories (Woburn, MA). Stock concentrations of the compounds were prepared in sterile water (TNFα and phleomycin) or in dimethylsulfoxide (DMSO) (5-FU, CPT, oxaliplatin, and erlotinib), and stored at -40°C, except TNFα, which was stored at -80°C. Antibodies against p65, NF-κB, cIAP2, and Bcl-X_L_ were purchased from Cell Signaling Technology (Danvers, MA), and anti-tubulin (M2) antibody from Sigma Aldrich. SignalSilence® NF-κB p65 siRNA I (#6261) was purchased from Cell Signaling Technology and NF-κB inhibitor III (SM7368) from EMD Millipore (Billerica, MA). The Chk1/Chk2 specific inhibitor AZD-7762 was purchased from Sigma Aldrich (St. Louis, MO).

### Generation and testing of NF-κB reporter SW480 and HCT116 cells

NF-κB reporter stable cells were established by transducing p53-mutant SW480 (ATCC), p53 wild-type HCT116, and p53-null HCT116 (both from Dr. Vogelstein) colon cancer cells with lentiviral constructs containing NF-κB transcriptional response elements (TREs) linked to the luciferase gene (Qiagen, Valencia, CA). In parallel, cells transduced with a construct that lacks the TREs, and which therefore do not respond to NF-κB activation, were used as negative controls to validate the specificity of reporter activity. A construct expressing GFP was used to assess transduction efficiency, which was 100 percent. Transduced cells were selected in a medium containing puromycin (2.5 μg/ml), a concentration established to kill 100% of the control cells within 3 days. To minimize any insertion site bias, pooled populations of transduced cells were used for the assays.

### Luciferase assays

For luciferase assays, cells were seeded and treated in 96-well plates. Before reading the plates, the culture medium was removed by aspiration, and 50 μL of 1× luciferin-PBS substrate solution was added to each well. With a luminometer set at 37°C, plates were read immediately after addition of substrate solution and after 5 and 10 minutes. The time point at which peak readings for all the wells were obtained was taken for calculation of relative luciferase units (RLU). Luciferase expression was quantified as RLU, normalized to readings of control wells, and expressed as relative NF-κB reporter activity.

### Cytokine assays

Colorimetric CXCL8 and IL-6 ELISA kits were purchased from R&D Systems, and the assays were performed according to the manufacturer’s instructions. Culture supernatants from equivalent numbers of cells seeded in multi-well plates were harvested 24 hours after the last treatment. Total protein in the supernatants was measured with DC Protein Assay (BioRad, Herculus, CA) and volume-adjusted with sterile PBS to the sample with the lowest protein content. Samples were diluted 1:3 in the assay diluent buffer. Color development at the end of ELISA assays was measured with a microplate reader (BioTek, Winooski, VT).

### RT-PCR for cytokine and receptor gene expression analysis

Total RNA was extracted from cells by use of RNeasy extraction kits (Qiagen, Valencia, CA). QuantiTect cDNA synthesis kits (Qiagen) were used to reverse transcribe 100 ng of RNA in a final volume of 20 μL. RNA and cDNA were stored at -80°C until used. Primers suitable for RT-PCR were designed using the PrimerQuest designer tool (IDT DNA, Coralville, IA), ensuring exon spanning. Primer sequences in 5′ to 3′ orientation were: CXCL8 forward, CTTGGCAGCCTTCCTGATTT, reverse, GGGTGGAAAGGTTTGGAGTATG; CXCR1 forward, CAAGTGCCCTCTAGCTGTTAAG, reverse, CAGCAATGGTTTGATCTAACTGAAG; CXCR2 forward, CATCGTCAAGGTTGTTTCATCTT, reverse, AGCTGTGACCTGCTGTTATT; and IL6 forward, AAAGAGGCACTGGCAGAAA, reverse, CAGGCAAGTCTCCTCATTGAA. SYBR Green PCR was performed by use of Quantitect SYBR Green master mix (Qiagen) and run on a MX3005P or MX3000P thermocycler from Agilent Technologies/Stratagene (Santa Clara, CA). For each experiment, expression values were normalized against the control values.

### CellMiner data mining and analysis

CellMiner tool (http://discover.nci.nih.gov/cellminer/home.do; version 1.5) was used to compare and plot the relative baseline expression of CXCR1 and CXCR2 mRNA among colon cancer cells included in the NCI-60 panel. The tool enables retrieval and integrated analysis of baseline and experimental data compiled from the 60 cell lines included in the panel [34, 35]. CellMiner gene transcript data was generated from five microarray platforms. To generate the transcript graph for colon cancer cells, we selected gene transcript level z-score for analysis type and CXCR1 and CXCR2 as gene identifier inputs.

### Immunofluorescence staining

Cells for immunofluorescent staining were grown and treated in chamber slides, and then fixed in 4% formaldehyde in PBS for 10 minutes, permeabilized for 10 minutes with 0.2% Triton X-100 in PBS, and blocked with 2% BSA for 1 hour. Rabbit primary antibody to p65 (Cell Signaling®) was diluted at 1:400 in PBS containing 1% BSA and incubated for 1 hour at room temperature. AF-488 anti-rabbit secondary antibody was from Life Technologies® (Grand Island, NY), and was diluted 1:250 in 1% BSA in PBS, and incubated for 1 hour. Images were captured using Olympus® BX53 optical microscope.

## Results

### Signal-specific response of reporter cells

**Figure 1:**
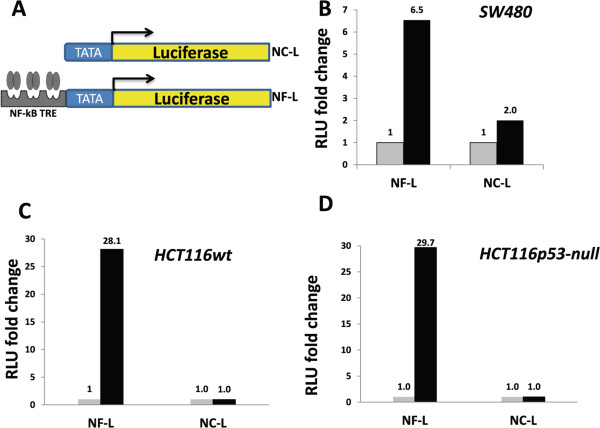
**Generation and testing of NF-κB reporter cells. A**: schematic representation of NF-κB reporter constructs; NC-L = negative control luciferase, NF-L = NF-κB luciferase. **B**, **C**, and **D**: relative NF-κB activity indices for reporter SW480, wild type HCT116, and p53-null HCT116 colon cancer cells, respectively. Grey bars = unstimulated; black bars = TNF-stimulated (at 100 ng/ml concentration).

**Figure 2:**
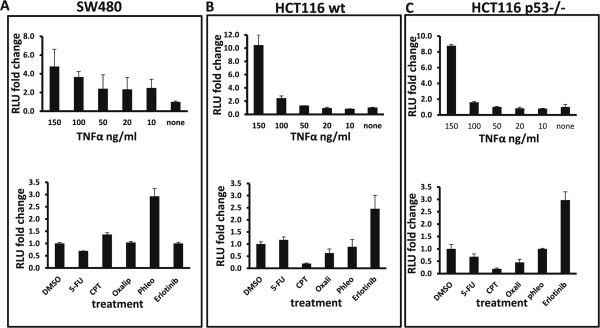
**Dependence of NF-κB response on type and dose of treatment. A-C** (upper panels): Reporter SW480, wild-type HCT116, and p53-null HCT116 colon cancer cells were treated with decreasing concentrations of the cytokine, TNFα. The activation of NF-κB is presented as fold change in RLU compared to the control (none). **A-C** (lower panels): The reporter cells were treated with vehicle (DMSO), 5-FU (10 μM), CPT (1 μM), oxaliplatin (10 μM), phleomycin (200 μg/ml), or erlotinib (20 μM). The NF-κB activation index at 24 hours after treatment is shown. The cell lines responded differently to these clinically relevant drugs.

To assess the activation of NF-κB in response to drugs that are clinically used to treat colon cancer, NF-κB reporter cells were established by lentivirus-mediated transduction of a construct made of NF-κB response elements fused to the luciferase gene. First, the reporter cells were tested to see if they responded to NF-κB pathway activation by treating them with the NF-κB inducer, TNFα. Cells transduced with NF-κB TRE, but not those transduced with a construct without the TRE, were responsive to treatment with TNFα (Figure 1). Further, parental HCT116 cells, which are wild-type for p53, responded similarly to the isogenic p53-null HCT116 cells, suggesting that the absence of p53 in these cells did not affect the activation of NF-κB by TNFα. To validate these results, concentration-dependent NF-κB activation by TNFα was examined. Ranges of 10–150 ng/ml TNFα induced NF-κB activation in a concentration-dependent manner (Figure 2A-C upper panels). Again, wild-type and p53-null HCT116 cells responded similarly, strengthening the observation that, in this context, the absence of p53 does not influence the activation of NF-κB.

### Drug- and cell type-dependent NF-κB responses in SW480 and HCT116 colon cancer cells

Having established the responsiveness of these cells to NF-κB pathway activation, the effects of four drugs currently in clinical use, 5-FU (10 μM), CPT (1 μm), oxaliplatin (10 μM), and erlotinib (20 μM), as well as phleomycin (100 μg/ml), a radiomimetic compound, were determined. Among these, only erlotinib (an EGFR inhibitor) is a receptor-targeted drug; the others are non-selective. The results (Figure 2A-C, lower panels) show that SW480 and HCT116 cells respond to these drugs differently. The radiomimetic drug phleomycin induced the highest activation of NF-κB reporter activity in the p53 mutant SW480 cells, but only erlotinib induced NF-κB in both wild-type and p53-null HCT116 cells. As in the previous results, there was no difference in the pattern of NF-κB activation between the p53-null and wild-type HCT116 cells. However, unlike in SW480 cells, CPT decreased the level of basal reporter activity in both types of HCT116 cells. In contrast, CPT treatment consistently increased the activation of NF-κB reporter activity in SW480 cells, albeit to a lower extent relative to phleomycin. 5-FU and oxaliplatin did not induce remarkable activity in these cell lines and therefore were not utilized further (Additional file 1: Figure S1).

### Concentration-dependent NF-κB response in SW480 and HCT116 colon cancer cells

**Figure 3:**
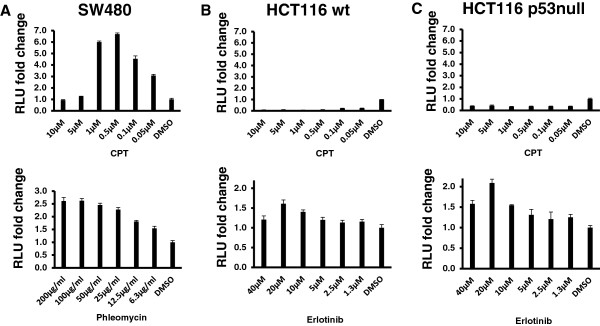
**Drug concentration-dependent NF-κB response in colon cancer cells.** SW480 **(panel A)**, wild-type HCT116 **(panel B)**, or p53-null HCT116 **(panel C)** cells were treated with the indicated concentrations of CPT, phleomycin (SW480 only), or erlotinib (HCT116 only) for 24 hours, and reporter assays were performed. The relative NF-κB activity indices (Y-axis) relative to vehicle (DMSO control), plotted against the drug concentrations (X-axis), are shown.

Visual examination of HCT116 cells treated with CPT at concentrations of 0.5 μM or above showed increased death, which suggested that these cells are relatively sensitive to the drug. This raised the possibility that the decrease in reporter activity may be due to the loss in cell viability. To rule out this effect, equivalent numbers of SW480 and HCT116 cells were exposed to varying concentrations of CPT, phleomycin, and erlotinib, and NF-κB reporter activity was measured. Whereas SW480 cells showed a modal response peaking at 0.5 μM CPT and decreasing at either higher or lower concentrations, HCT116 cells did not report any NF-κB activity even at 0.05 μM CPT, the lowest concentration tested (Figure 3A-C). For concentrations at and below 0.1 μM, there was no evident loss of cell viability for any of the cells within the first 24 hours, ruling out the possibility that loss in viability reduced reporter activity. For SW480 cells, concentrations of 5 μM and above reduced cell viability, explaining the drop in reporter activity at those concentrations (Figure 3A). At a concentration of 20 μM, erlotinib induced the highest reporter activity in both wild-type and p53-null HCT116 cells, but there was no loss of viable cells at the concentrations tested. Phleomycin induced the highest reporter activity in SW480 cells at 100 μg/ml, an effect that remained unchanged at 200 μg/ml. The results show that NF-κB activation by these drugs varies based on the cell types, drug types, and concentrations used. Although the drugs used in these experiments are clinically relevant, we decided to further examine the activation of NF-κB by CPT only, because this drug is widely clinically used and it gave consistently higher NF-κB response at sub-micromolar concentrations. Moreover, phleomycin is only radiomimetic, and unlike EGFR inhibition by monoclonal antibodies, EGFR inhibition by tyrosine kinase inhibitors (TKI) such as erlotinib in colon cancer has not achieved wide clinical utility [36].

### NF-κB activation by CPT is accompanied by p65 nuclear re-localization

**Figure 4:**
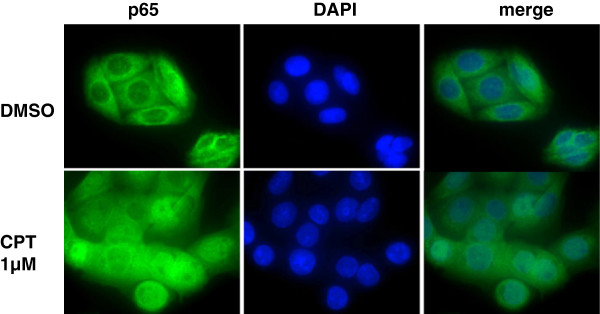
**Nuclear redistribution of p65 NF-κB in response to CPT in SW480 colon cancer cells.** Parental SW480 cells were treated with 1 μM CPT, a concentration that induces reporter activity. Translocation of the p65 protein, a marker for the activation of the canonical NF-κB pathway, was examined by immunofluorescent staining of control (DMSO, upper row) and CPT-treated (lower row) cells. CPT induces partial translocation of the p65 protein.

The canonical pathway for NF-κB activation involves the re-localization of NF-κB p65-p50 dimers to the nucleus [37]. To determine if the activation of NF-κB by CPT involves such a re-distribution, parental SW480 cells were treated with CPT (1 μM), and the intracellular localization of the p65 subunit was detected by immunofluorescent staining using a p65 antibody. Treatment of the cells with CPT for 24 hours resulted in re-distribution of the p65 protein to the nuclear compartment (Figure 4), indicating the involvement of p65 and its dimers in the NF-κB response to the drug.

### NF-κB activation by CPT in SW480 cells is accompanied by up-regulation of CXCL8, but not of cIAP2 or Bcl-X_L_

**Figure 5:**
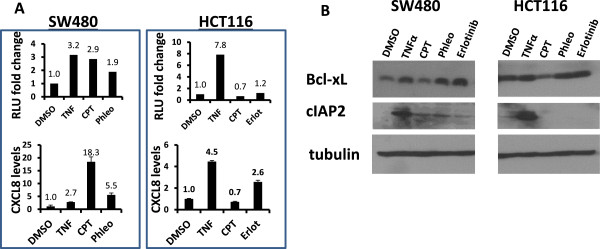
**Differential NF-κB pathway activation in response to CPT treatment of SW480 cells. A**: Reporter SW480 or HCT116 cells were treated with vehicle (DMSO) or with TNFα (100 ng/ml), CPT (1 μM for SW480 and 0.05 μM for HCT116 cells), phleomycin (SW480 only, 100 μg/ml), or erlotinib (HCT116 only, 20 μM). Upper panels show relative NF-κB reporter activity (Y-axis) plotted against the treatments (Y-axis); lower panels show relative amounts of secreted CXCL8 in culture supernatants from treated cells. **B**: In SW480 and HCT116 reporter cells, the NF-κB target genes, cIAP2 or Bcl-X_L_, are up-regulated by TNFα and other drugs, but not by CPT. Parental SW480 or HCT116 cells were treated with the indicated drugs as in panel A, and the expression of Bcl-X_L_ and cIAP2 was analyzed by immunoblotting. Tubulin bands are shown as a loading control.

Signaling through the NF-κB pathway regulates genes involved in various cellular processes, including inflammation, apoptosis, cell survival, motility, invasion, and resistance to drugs. Therefore, pathways potentially activated by treatment of SW480 cells with CPT were assessed. The expressions of two cytokines (CXCL8 and IL-6) and two anti-apoptotic genes (*cIAP2* and *Bcl-X*_*L*_), all of which are downstream targets of the NF-κB pathway, were examined. TNFα, an inducer of the NF-κB pathway, was used as a control. TNFα induced reporter activity, cytokine secretion, and *cIAP2* gene expression in both SW480 and HCT116 parental cells, suggesting similar NF-κB response mechanisms in both types of cells (Figure 5A-B). In contrast, CPT treatment of SW480 cells up-regulated the reporter activity and cytokine secretion, but not *cIAP2* gene expression (Figure 5A-B left panels). Consistent with these results, CPT did not increase the reporter activity nor up-regulate CXCL8 or cIAP2 in HCT116 cells (Figure 5A-B right panels). Erlotinib also increased the secretion of CXCL8 in HCT116 cells, consistent with its activation of NF-κB reporter activity in these cells. Since IL-6 in the culture supernatants of treated or untreated SW480 and HCT116 cells could not be detected by ELISA, there was no further examination of CPT mediated IL-6 response. These results provide evidence for uncoupling of drug-induced NF-κB activity from the suppression of apoptosis through increased anti-apoptotic gene expression by NF-κB. In these experiments, reporter cells were used to generate data for reporter activities, but gene expression and protein assays were accomplished with parental cells.

### Chemical and molecular inhibition of the NF-κB pathway suggests cytokine induction by CPT proceeds through alternative mechanisms

**Figure 6:**
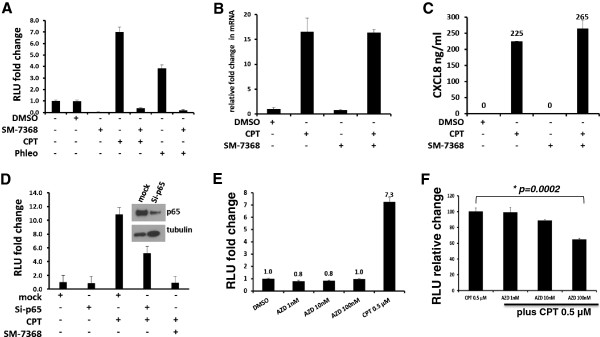
**Effect of chemical or molecular inhibition of NF-κB on CXCL8 secretion by SW480 cells in response to CPT. A**: Reporter SW480 cells were left untreated or treated with DMSO (vehicle), NF-κB inhibitor SM7368 (10 μM), CPT (1 μM), phleomycin (100 μg/ml), or combinations of CPT or phleomycin with SM7368. Relative NF-κB reporter activity was measured by luciferase reporter assay. **B-C**: Parental SW480 cells were treated with DMSO (vehicle), CPT, SM7368, or a combination of CPT and SM7368 as shown and at the same concentrations as in A. The expression of CXCL8 was analyzed at mRNA level by qRT-PCR **(B)** or by cytokine ELISA in cell culture supernatant **(C)**. Relative expressions in comparison to that from vehicle treated cells are indicated. **D**: Reporter SW480 cells were transfected with control (mock) or specific siRNA against p65 (si-p65) for 24 hours, after which they were treated with DMSO (vehicle) or CPT. NF-κB reporter activity was measured 24 hours after treatment. Cells were also treated with CPT in combination with SM-7368 for comparison. **E-F**: Effects of Chk1/Chk2 inhibition on NF-κB activity. NF-κB reporter SW480 cells were treated with varying concentrations (0–100nM) of the Chk1/Chk2 inhibitor AZD-7762 or 0.5 μM CPT as single agents **(E)** or varying concentration of AZD-7762 in combination with 0.5 μM CPT **(F)**. NF-κB activation was measured by luciferase assay. While AZD-7762 by itself did not have an effect on NF-κB activation, it significantly (student’s *T*-test, *p* = *0.0002*) inhibited CPT-induced NF-κB activation at100 nM concentration.

The observation that cytokine secretion was not concomitant with cIAP2 up-regulation prompted us to determine if chemical inhibition of NF-κB or inhibition by siRNA against the p65 subunit eliminated the reporter activity and the secretion of CXCL8 in CPT-treated SW480 cells. To this end, 1 μM CPT, alone or in combination with 10 μM SM-7368 (an inhibitor of NF-κB activation, Millipore), was used to measure NF-κB activity in reporter SW480 cells and CXCL8 mRNA and protein expressions in parental SW480 cells. The combination of SM-7368 with either CPT or phleomycin suppressed NF-κB reporter activity (Figure 6A). However, the inhibitory effect of SM-7368 on the reporter activity did not result in the reduction of CXCL8 at the mRNA and protein levels (Figure 6B-C), suggesting that the mechanisms of CXCL8 secretion in SW480 cells treated with CPT involves regulatory factors beyond NF-κB response elements in the promoter of CXCL8.

Since SM-7368 may interfere with NF-κB activation at different levels in the signaling network, leading to broader inhibition, we determined if inhibition of NF-κB by siRNA-mediated reduction of p65 expression would prevent activation of the NF-κB pathway induced by CPT. Reporter SW480 cells were first transfected with p65 siRNA and, 24 hours later, treated with 1 μM CPT. At 24 hours after the treatment, cells were assayed for reporter activity. Reduction of p65 levels was accompanied by a decrease (up to 50%) in the reporter activity (Figure 6D), showing that, after treatment of cells with CPT, p65-dependent and -independent mechanisms may be involved in the activation of NF-κB. Since CPT induces DNA damage as a mechanism of action, we then examined if chemical inhibition of DNA damage signaling through Chk1/Chk2 kinases would interfere with CPT-induced NF-κB activation. To test this possibility, we treated SW480 NF-κB reporter cells with the Chk1/Chk2 specific inhibitor AZD-7762 or CPT as single agents, or in combination. As shown in Figure 6E-F, while the Chk1/Chk2 inhibitor alone showed no effect on the basal NF-κB reporter activity, it significantly inhibited the NF-κB activation induced by CPT.

### CPT treatment up-regulates the expression of CXCL8 receptors CXCR1 and CXCR2 in HCT116 colon cancer cells

**Figure 7:**
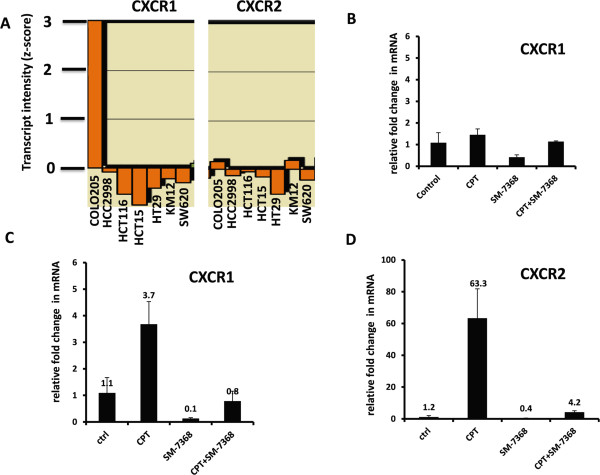
**Changes in CXCR1 and CXCR2 expression induced by CPT treatment. A**: Negligible basal expression of CXCR1 and CXCR2 in HCT116 and SW620 cells (Cell Miner [36]). **B**: Relative fold change in CXCR1 mRNA in SW480 cells treated with CPT, SM7368, or a combination of the two. **C-D**: Relative fold changes in mRNAs for CXCR1 and CXCR2 in HCT116 cells treated with vehicle (ctrl), CPT (0.05 μM), SM7368 (10 μM), or a combination of the two. CPT induced a marked increase in CXCR2 expression in HCT116 cells, and the expression was inhibited by treatment of the cells with SM7368, an NF-κB inhibitor.

The cytokine/chemokine CXCL8 mediates signal transduction through two G-protein coupled receptors, CXCR1 and CXCR2. It has been proposed that CXCL8 functions as a praracrine or autocrine regulator of various cell functions, including enhanced cell motility and cell survival [38]. Therefore, expression of these two CXCL8 receptors was examined by RT-PCR in SW480 and HCT116 cells before and after treatment with CPT (1 μM for SW480 and 0.05 μM for HCT116 cells) for 24 hours. From the cells, mRNA was harvested, and 100 ng was reverse transcribed to cDNA. The relative expression of these genes was analyzed by SYBRgreen real-time PCR. Examination of the basal levels of CXCR1 and CXCR2 expression in the NCI-60 panel of colon cancer cell lines using the genomic and pharmacologic tool Cell Miner [35] showed that HCT116 cells and SW620 cells (derived from metastasis of SW480) express negligible or no CXCR1 or CXCR2 (Figure 7A), and CPT treatment did not induce changes in the mRNA expression of CXCR1 in SW480 cells (Figure 7B). The expression of mRNA for CXCR2 in both untreated and CPT-treated SW480 cells remained below the threshold for detection by RT-PCR and therefore is not shown. HCT116 cells, which failed to activate NF-κB and secrete CXCL8 in response to CPT, showed a robust increase in the expression of both CXCL8 receptors CXCR1 and CXCR2 (Figure 7C-D), particularly that of CXCR2. This response was confirmed by two additional experiments where, in each case, CXCR2 expression in HCT116 cells treated with 0.05 μM CPT was consistently high. The increase in the expression of CXCR1 remained moderate (Figure 7C). The up-regulation of CXCR1 and CXCR2 by CPT in HCT116 cells was inhibited by SM-7368, a chemical inhibitor of NF-κB activation.

## Discussion

The evidence presented here indicates that treatment of colon cancer cells with broad-acting and targeted chemotherapeutic drugs leads to heterogeneous responses that vary depending on the cellular make-up and the type of drug used. Adding to the complexity of such responses, no comparable NF-κB response was evident, even when drugs with similar known mechanisms of action (for example, DNA damage) were used on colon cancer cells, and neither did the same drug elicit similar responses in different types of cells. While the response of cells to a given drug could be dynamic, identification of the factors that determine which cells will respond to a given drug by activating the NF-κB pathway emerges as a new challenge. Moreover, given the heterogeneity of cells in tumor tissues and their microenvironments, the question of which of these cells exposed to chemotherapeutic drugs or radiation respond in a particular way needs to be addressed. Such responses include secretion of proteins that regulate motility, vasculature, drug resistance, cytokines, and growth factors as well as the receptors for those factors. Moreover, the functions of such predictable or dynamic responses to the outcomes of cancer treatment remain challenges to be addressed.

The activation of NF-κB in response to chemotherapy is established [25, 39, 40], although the mechanisms and the functions of such activation remain largely unknown. Inhibition of NF-κB activation may sensitize cells to CPT [41, 42]. NF-κB pathways could be activated through two mechanisms: signals that originate at cell receptors and signals that originate in the nucleus [10, 37]. The pathways that originate at the cell membrane involve the TNF receptor-family proteins as well as their downstream adaptor and signal transducer proteins [37, 43]. Nevertheless, the nuclear signaling of NF-κB activation is still largely unknown. Nuclear-mediated activation of NF-κB involves DNA-damage proteins, primarily the ATM/ATR kinase proteins, which transduce the signal to the cytoplasm through the adapter protein, NEMO [24, 25, 32]. It is perplexing that not all DNA-damaging drugs activate NF-κB in colon cancer cells, even under similar conditions. Since cell lines vary from one another, identification of key regulators for nuclear NF-κB activation and systematic examination of their functions could elucidate the mechanisms behind the activation. Moreover, the activation of NF-κB by receptor-acting erlotinib only in HCT116 cells raises another level of complexity, because both SW480 and HCT116 cells are wild-type for the erlotinib target, EGFR. It is possible that erlotinib has targets that are differentially expressed between SW480 and HCT116 cells, or that signaling intermediates downstream of EGFR may be divergent in cross-talk with the NF-κB pathway.

Perhaps activation of specific genes by NF-κB requires interactions with additional regulatory factors. For example, the CXCL8 promoter contains AP1 transcription factor binding sites that may co-regulate expression of the cytokine [44]. Accordingly, cross-talk between the AP-1 and NF-κB pathways may explain the differential regulation of CXCL8 and anti-apoptotic proteins downstream of NF-κB activation. Further studies are needed to discern how this distinction is achieved in cells.

The up-regulation of CXCR2 and CXCR1 receptors by HCT116 cells, which do not activate NF-κB in response to CPT, raises the possibility that subgroups of cells in a heterogeneous tumor mass may, under chemotherapy, secrete or respond to soluble factors in the microenvironment. Although HCT116 and SW480 cells are more evolutionarily divergent from each other than cancer cells in a patient, the heterogeneity in solid tumors and their metastases does not preclude the existence of subsets of cells with different secretory and responsive characteristics. Therefore, it is rational to suggest that the combination of CPT therapy with antagonists of CXCR2 and CXCR1, especially in individuals who respond to CPT by activation of NF-κB, may improve the therapeutic efficacy. To enhance the efficacy of chemotherapy, further studies are needed to identify additional targets in the NF-κB – CXCR2/CXCR1 axis.

## Conclusion

In response to cancer therapeutic agents, NF-κB activation varies with the cellular make up and that drug-induced NF-κB activation may be functionally uncoupled from anti-apoptotic outcomes found for other stimuli. Some cancer cells in a heterogeneous tumor tissue may, under therapeutic pressure, release soluble factors that have paracrine activity on neighboring cells that express the cognate receptors. The potential benefits of targeting these soluble factors and their receptors alongside mainstream chemotherapy need to be further studied.

## Electronic supplementary material

Additional file 1: Figure S1: Effects of anti-colon cancer chemotherapeutic drugs on NF-κB reporter activity. NF-κB reporter HCT116 or SW480 cells were treated with CPT, 5-FU, or oxaliplatin at the concentrations shown for 24 hours after which NF-κB activation was measured by luciferase assay. Results show that NF-κB was strongly activated only in SW480 cells by CPT in low micromolar ranges, whereas 5-FU in concentrations above 10 uM induced moderate NF-κB response, but only in HCT116 cells. Y-axis represents raw luciferase assay luminescence units readings. (PPTX 96 KB)

## Abbreviations

CPT: Camptothecin
NF-κB: Nuclear factor kappa-light-chain-enhancer of activated B cells
CXCR: C-X-C chemokine receptor
EGFR: Epidermal growth factor receptor
cIAP2: Cellular inhibitor of apoptosis 2.
