# Supplementary material for: Variable NF-κB pathway responses in colon cancer cells treated with chemotherapeutic drugs
Source: BMC Cancer. 2014 Aug 18;14:599. doi: 10.1186/1471-2407-14-599 (PMC4152571; doi:10.1186/1471-2407-14-599)
Supplement: Supplementary file 1 — Additional file 1: Figure S1: Effects of anti-colon cancer chemotherapeutic drugs on NF-κB reporter activity. NF-κB reporter HCT116 or SW480 cells were treated with CPT, 5-FU, or oxaliplatin at the concentrations shown for 24 hours after which NF-κB activation was measured by luciferase assay. Results show that NF-κB was strongly activated only in SW480 cells by CPT in low micromolar ranges, whereas 5-FU in concentrations above 10 uM induced moderate NF-κB response, but only in HCT116 cells. Y-axis represents raw luciferase assay luminescence units readings. (PPTX 96 KB) [file 12885_2014_4792_MOESM1_ESM.pptx]

## Slide 1
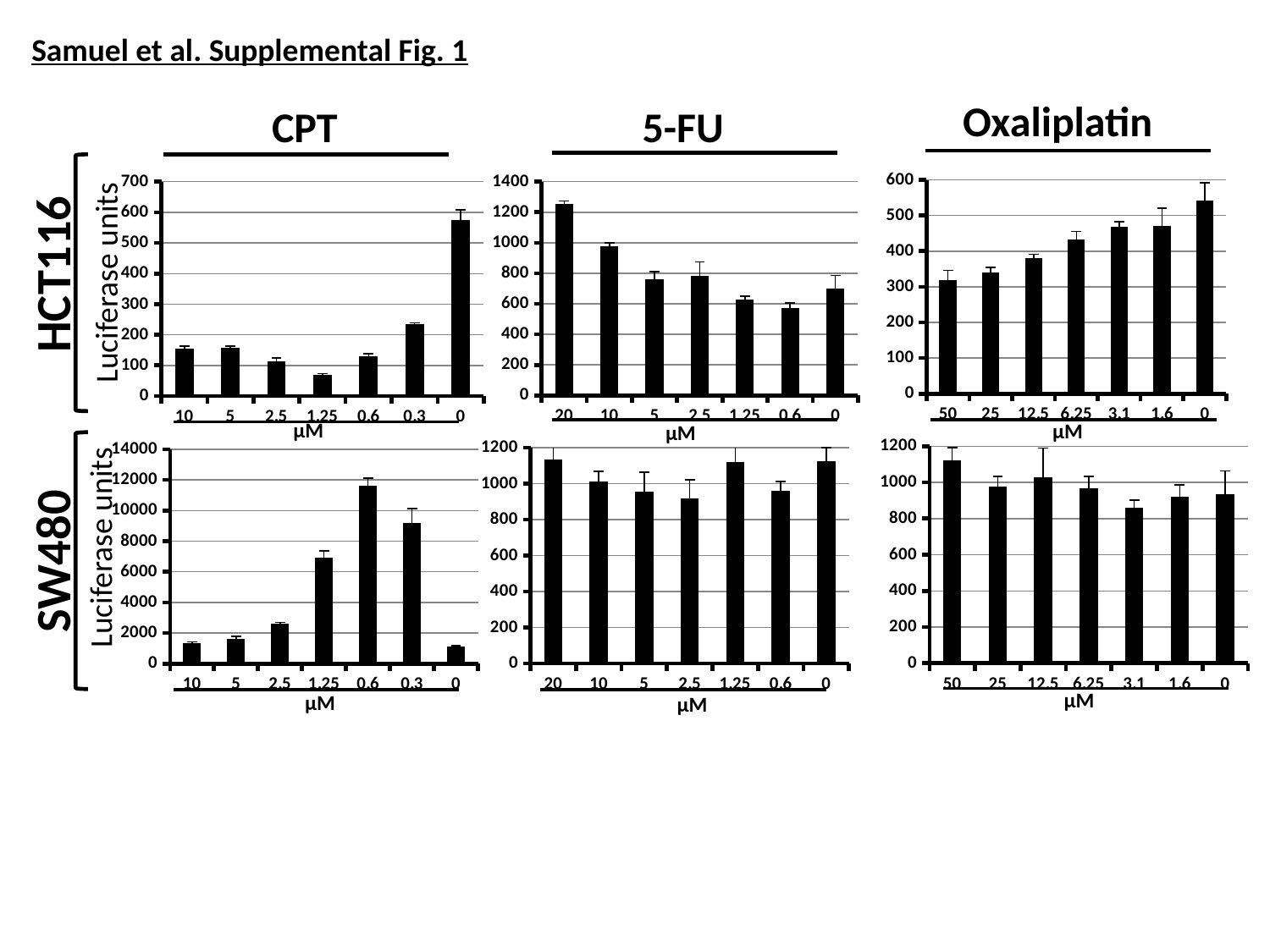

Samuel et al. Supplemental Fig. 1
Oxaliplatin
CPT
5-FU
### Chart
| Category | |
|---|---|
| 50 | 317.6666666666667 |
| 25 | 339.0 |
| 12.5 | 380.0 |
| 6.25 | 431.6666666666667 |
| 3.1 | 468.6666666666667 |
| 1.6 | 471.0 |
| 0 | 541.3333333333334 |
### Chart
| Category | |
|---|---|
| 10 | 154.0 |
| 5 | 156.0 |
| 2.5 | 114.0 |
| 1.25 | 68.33333333333333 |
| 0.6 | 128.33333333333334 |
| 0.3 | 234.33333333333334 |
| 0 | 576.0 |
### Chart
| Category | |
|---|---|
| 20 | 1251.6666666666667 |
| 10 | 974.6666666666666 |
| 5 | 759.0 |
| 2.5 | 782.6666666666666 |
| 1.25 | 630.3333333333334 |
| 0.6 | 574.6666666666666 |
| 0 | 699.6666666666666 |HCT116
### Chart
| Category | |
|---|---|
| 50 | 1123.0 |
| 25 | 977.0 |
| 12.5 | 1029.0 |
| 6.25 | 967.0 |
| 3.1 | 860.3333333333334 |
| 1.6 | 921.3333333333334 |
| 0 | 933.3333333333334 |
### Chart
| Category | |
|---|---|
| 20 | 1133.3333333333333 |
| 10 | 1012.6666666666666 |
| 5 | 955.6666666666666 |
| 2.5 | 917.3333333333334 |
| 1.25 | 1121.6666666666667 |
| 0.6 | 961.0 |
| 0 | 1124.6666666666667 |
### Chart
| Category | |
|---|---|
| 10 | 1365.6666666666667 |
| 5 | 1642.3333333333333 |
| 2.5 | 2619.0 |
| 1.25 | 6938.0 |
| 0.6 | 11619.666666666666 |
| 0.3 | 9192.666666666666 |
| 0 | 1096.0 |SW480
µM
µM
µM
µM
µM
µM
Luciferase units
Luciferase units
